# Relationship between anthropometric parameters and open angle glaucoma: The Korea National Health and Nutrition Examination Survey

**DOI:** 10.1371/journal.pone.0176894

**Published:** 2017-05-08

**Authors:** Jae Yeun Lee, Tae-Woo Kim, Hyun Tae Kim, Mi Yeon Lee, Hye Won Min, Yu Sam Won, Hyun Seok Kwon, Ki Ho Park, Joon Mo Kim, on behalf of the Epidemiologic Survey Committee of the Korean Ophthalmological Society

**Affiliations:** 1 Department of Ophthalmology, Kangbuk Samsung Hospital, Sungkyunkwan University School of Medicine, Seoul, Republic of Korea; 2 Department of Ophthalmology, Seoul National University Bundang Hospital, Seoul National University College of Medicine, Gyeonggi-do, Republic of Korea; 3 Division of Biostatistics, Department of R&D Management, Kangbuk Samsung Hospital, Sungkyunkwan University School of Medicine, Seoul, South Korea; 4 Division of Medical Information, Department of Physician Education & Training, Kangbuk Samsung Hospital, Sungkyunkwan University School of Medicine, Seoul, Republic of Korea; 5 Department of Neurosurgery, Kangbuk Samsung Hospital, Sungkyunkwan University School of Medicine, Seoul, Republic of Korea; 6 SU Yonsei Eye Clinic, Seoul, Republic of Korea; 7 Department of Ophthalmology, Seoul National University Hospital, Seoul National University College of Medicine, Seoul, Republic of Korea; Soochow University Medical College, CHINA

## Abstract

**Aims:**

To evaluate the relationships between open-angle glaucoma (OAG) and various anthropometric measurements.

**Design:**

Korea National Health and Nutrition Examination Survey (KNHANES), a population-based cross-sectional study using a complex, stratified, multistage, probability-cluster survey.

**Methods:**

A total of 5,255 participants including 247 glaucoma patients, aged ≥ 19 years were included from the KNHANES V database. Glaucoma diagnosis was based on International Society of Geographical and Epidemiological Ophthalmology criteria. Various anthropometric data regarding obesity were analyzed including body mass index (BMI), total body fat mass, total body muscle mass (lean body mass, non-bone lean body mass, and appendicular skeletal muscle (ASM) mass), and waist circumference (WC). The differences in OAG prevalence with respect to anthropometric parameter quartiles were examined.

**Results:**

In males, the multivariate general linear model adjusted for age, alcohol, smoking, exercise, systemic hypertension, diabetes, and intraocular pressure (IOP) showed the quartiles for the anthropometric parameters BMI, fat mass/weight ratio and fat mass/muscle mass ratio were negatively associated with OAG. However, muscle mass parameter/BMI ratio was significantly positively associated with OAG (P for trend<0.05). In females, height and fat mass/BMI showed a significant relationship with the risk of OAG. (P value<0.05)

**Conclusions:**

In the present study, high fat mass was associated with low OAG risk. Body composition seemed to affect the prevalence of OAG, but further evaluation is needed.

## Introduction

Glaucoma is considered a multifactorial disease and is associated with demographic factors including age, gender, and race, as well as ocular and systemic factors. Recently, several studies have reported that metabolic syndrome was associated with open-angle glaucoma (OAG) [1,2]; however, the association has not been consistent, and the pathophysiology remains unclear [3–5].

Obesity is considered an important risk factor for metabolic syndromes such as type 2 diabetes mellitus or coronary heart disease [6,7]. Body mass index (BMI) is a popular index used to estimate obesity. The World Health Organization (WHO) defines obesity as a BMI of 30 kg/m^2^ or greater and overweight as individuals with BMIs between 25 kg/m^2^ and 29.9 kg/m^2^ [8]. High BMI is considered a factor strongly related to the increasing prevalence of metabolic syndromes [9]. Several studies have reported a relationship between BMI and ocular disease [5,10,11]. However, whether BMI is the best parameter for estimating obesity is currently unclear. The body consists of various tissues such as fat, muscle, bone, and soft tissue; however, BMI is calculated using only body weight and height. Furthermore, the effect of each body component on ocular physiology and disease remains unclear. Several studies have suggested that abdominal obesity rather than BMI is a better surrogate for metabolic disease [12,13].

Dual-energy X-ray absorptiometry (DEXA) can accurately detect adiposity and provide information regarding total and regional fat mass and lean body mass and bone mineral contents [14]. In this study, we investigated the association between not just BMI, but various anthropometric variables and OAG development to determine the effect of each body component using nationally representative data for South Korean adults extracted from the Korea National Health and Nutrition Examination Survey (KNHANES).

## Materials and methods

This study adhered to the tenets of the Declaration of Helsinki for human research, and all participants provided written informed consent. The survey protocol was approved by the Institutional Review Board of the Korea Center for Disease Control and Prevention (KCDCP). Because annually, KCDCP published the reports and microdata of KNHANES with survey manuals through the official website of KNHANES (http://knhanes.cdc.go.kr), all KNHANES data is de-identified and available to the public, the Institutional Review Board of Kangbuk Samsung Hospital determined that this study was exempt from requiring their approval.

### Study design and population

The KNHANES is an ongoing, population-based, cross-sectional survey in South Korea conducted by the KCDCP and the Korean Ministry of Health and Welfare. It uses a multistage, stratified, probability-cluster survey with a rolling sampling design. Therefore, the KNHANES is representative of the civilian, non-institutionalized South Korean population. The detailed design of the KNHANES has been previously described [15,16].

A total of 17,476 subjects were enrolled in KNHANES V (2010–2011). In KNHANES V data, participant exact medical history was added including refractive surgery. We included subjects that were aged 19 years or older, underwent eye examination and DEXA. We excluded subjects with any missing data. Participants were excluded if they were pseudophakic or aphakic, had a history of cataract, retinal or refractive surgery, evidence of retinal detachment, signs of AMD or diabetic retinopathy on examination. Finally, 5,225 subjects (2,214 males and 3,041 females) were enrolled in this analysis, including 247 OAG subjects (134 males and 113 females).

### Ophthalmological examination

All ophthalmic examinations were performed by ophthalmologists. A slit lamp examination including assessment of peripheral anterior chamber depth (ACD) using the Van-Herick method was performed (Haag-Streit model BQ-900; Haag-Streit AG, Koeniz, Switzerland). Peripheral ACD > 1/4 peripheral corneal thickness based on the Van Herick method was defined as OAG. Fundus photographs were taken with a digital non-mydriatic fundus camera (TRC-NW6S; Topcon, Tokyo, Japan, and Nikon D-80 digital camera; Nikon, Tokyo, Japan). Intraocular pressure (IOP) was measured with a Goldmann applanation tonometer (GAT; Haag-Streit model BQ-900; Haag-Streit AG, Koeniz, Switzerland) once for each eye from right to left. Visual field testing was performed with frequency doubling technology (FDT; Humphrey Matrix; Carl Zeiss Meditec, Inc., Dublin, CA, USA) using the N-30-1 screening test. The test location was defined as abnormal if it was not identified after two attempts at a contrast level that identified 99% of the healthy population. If two different test locations were abnormal, a visual field defect was defined in that eye. FDT was administered to participants suspected of having glaucoma and who met any of the following criteria: (1) IOP ≥ 22 mm Hg; (2) horizontal or VCDR ≥0.5; (3) nonadherence to the ISNT rule (neuroretinal rim thickness in the following order by quadrant: inferior > superior > nasal > temporal); (4) presence of optic disc hemorrhage (DH); or (5) presence of a retinal nerve fiber layer (RNFL) defect. FDT was repeated if either the rate of fixation errors or the false positive rate was > 0.33, in which case the FDT was defined to be an invalid test for glaucoma classification.

### Definition of OAG and healthy groups

The definition of OAG was based on the International Society of Geographical and Epidemiological Ophthalmology (ISGEO) criteria and a previous study [17–19]. Patients were defined as OAG if an open angle was present (peripheral ACD > 1/4 corneal thickness) and if any one of the following category I or category II diagnostic criteria were met.

Category I criteria were applied to subjects with FDT perimetry results showing a fixation error and false positive error of 1 or less. Glaucoma-diagnostic criteria were (1) loss of neuroretinal rim with vertical or horizontal CD ratio of 0.7 or more or a CD ratio asymmetry of 0.2 or more (both values determined by ≥97.5th percentile for the normal KNHANES population); (2) presence of optic disc hemorrhage; or (3) presence of an RNFL defect. Additionally, the subjects had to show abnormal FDT testing results with at least 1 location of reduced sensitivity compatible with optic disc appearance or RNFL defect. Criteria II were applied to those with absence of FDT perimetry results, fixation error or a false-positive error of 2 or more with (1) loss of neuroretinal rim with vertical CD ratio of 0.9 or more or asymmetry of vertical CD ratio of 0.3 or more (both values determined by ≥99.5th percentile for the normal KNHANES population) or (2) presence of RNFL defect compatible with optic disc appearance.

Healthy subjects were those who met the following criteria in both eyes: (1) IOP ≤ 21 mmHg; (2) presence of an open angle (peripheral ACD > 1/4 corneal thickness); (3) non-glaucomatous optic disc (vertical and horizontal CDR < 0.7 and inter-eye difference of vertical and horizontal CDR < 0.2); (4) absence of optic DH or RNFL defect); and (5) optic disc not violating the neuroretinal rim thickness order of inferior>superior>nasal>temporal rule.

After preliminary grading, detailed grading was performed independently by another group of glaucoma specialists who were blind to the participants’ other information. Any discrepancy between the preliminary and detailed grading was adjudicated by a third group (two glaucoma specialists).

### Anthropometric measurements and body composition

Body-weight was measured to the nearest 0.1 kg, and height was measured to the nearest cm with bare feet. Body mass index (BMI) was calculated from body-weight and height (kg/m2).

Specially trained examiners conducted participant anthropometric measurements. Waist circumference (WC) was measured at the midpoint between the lower border of the rib cage and the iliac crest while subjects were standing.

Total and regional (i.e., arm and leg) body fat mass and lean mass were measured using whole-body DEXA (QDR 4500A fanbeam densitometer, Hologic Inc., Bedford, MA, USA) by well-trained technicians. We acquired the parameters including total and regional fat mass, lean body mass, non-bone lean body mass, bone mineral content, and appendicular skeletal muscle (ASM) from DEXA.

Lean body mass was calculated as total body mass minus total fat mass. Non-bone lean body mass was calculated as lean body mass minus bone mineral contents. ASM mass was calculated as the sum of non-bone lean body mass of the arms and legs, following the method of Heymsfield et al [20].

We also obtained data regarding ratio of body component composition including fat mass, lean body mass, non-bone lean body mass, bone mineral content, ASM, and weight. In addition, to determine the effect of each component on OAG development at the same weight or BMI, we analyzed the ratio of body composition, BMI and the ratios of fat mass and other body components except fat mass.

### Lifestyle variables

All participants were asked about their lifestyle including alcohol consumption, smoking status, and physical activity. Based on average alcohol intake per day in the month before the interview, participants were categorized as heavy drinkers (> 60 g/day in males, > 40 g/day in females, two or more times a week) or not. Participants were categorized as current smoker (more than 100 cigarettes over lifetime and current smoking status) or not. On the basis of responses to the International Physical Activity Questionnaire, participants were considered regular physical exercisers if they performed moderate exercise more than five times per week for longer than 30 min per session or performed vigorous exercise more than three times per week for longer than 20 min per session [21].

### Statistical methods

Statistical analysis was performed using the Statistical Package for the Social Sciences (SPSS version 21.0; IBM SPSS, Inc., Chicago, IL, USA) to account for the complex sampling design. Strata, sampling units, and sampling weights were used to obtain point estimates and standard errors (SEs) of the mean. All data analyses were performed using weighted data, and SEs of the mean of population estimates were calculated using Taylor linearization methods. Participant characteristics were summarized for the entire sample using means and SEs for continuous variables and frequencies, percentages, and SEs for categorical variables.

Baseline demographic information and clinical parameters were compared between the groups using Pearson’s Chi-square test for categorical variables and general linear models for continuous variables.

General linear models were used to examine the relationships between anthropometric parameters and OAG. For this model, we adjusted for age, heavy drinking, current smoking, regular physical exercise, hypertension, diabetes, and IOP. After dividing the healthy subjects into quartiles for each anthropometric parameter, we analyzed the relationships between OAG and anthropometric parameters for each quartile.

Logistic regression models were used to estimate the odd ratios (ORs) with 95% confidence intervals (95% CIs). After all subjects were divided into quartiles for each anthropometric parameter, differences in the presence of OAG with respect to the quartiles were estimated. Quartile 1 was used as the reference value. β-coefficient value and 95% CIs were obtained. Because of the body composition differences between males and females, we stratified our analyses based on gender and then adjusted for age. ORs and 95% CIs for OAG risk were also obtained. *P* values were two-tailed, and a P value <0.05 was considered statistically significant.

## Results

A total of 17,476 subjects participated in the KNHANES between 2010 and 2011. The number of subjects who underwent ocular examination was 15,932. Of this subset, 9,925 subjects underwent ocular examination satisfying the International Society of Geographical and Epidemiological Ophthalmology criteria. Among them, the number of subjects whose anthropometric measurements were obtained with DEXA, including BMI, waist circumference, fat mass, lean body mass, and ASM mass was 6585. Of these 6585, 1360 subjects were excluded. Pseudophakia and aphakia by slit-lamp examination were exclusion criteria for this study.

The numbers of subjects with pseudophakia and aphakia were 338 (right eye) and 339 (left eye). In addition, the number of subjects excluded due to a history of ocular surgery (determined by questionnaire) included cataract surgery (355), retina surgery (15), refractive surgery (212), cataract and retina surgery (8) and cataract and refractive surgery (1). The final exclusion criteria included retinal detachment or age-related macular degeneration, as determined by fundus photographs. The number of subjects with abnormal fundus photographs was 556 for the right eye and 560 for the left eye. Sixty-one subjects who had a history of stroke (which can affect visual field testing) were excluded. Some subjects met multiple exclusion criteria.

The characteristics of study subjects are presented in Table 1. The mean age was 42.48 years, and the proportion of male subjects was 43.3%. The prevalence of OAG was 6.05% in males and 3.72% in females (total, 4.70%). The prevalence of OAG was significantly different based on gender (P = 0.001). All anthropometric parameters were significantly different based on gender (Table 2). The mean values for BMI, WC, lean body mass, non-bone lean body mass, and ASM were significantly higher in males than in females, while mean fat mass was higher in females than in males.

**Table 1:** Baseline demographics of study subjects by gender.

Variables	Healthy men (n = 2080, 48.9%) (Mean (SE))	OAG men (n = 134, 60.0%) (Mean (SE))	P	Healthy women (n = 2928, 51.1%) (Mean (SE))	OAG women (n = 113, 40.0%) (Mean (SE))	P
**Age (years)**	40.97 (0.45)	48.98 (1.66)	<0.001	42.80 (0.39)	54.21 (2.23)	<0.001
**Intraocular pressure (mmHg)**	14.24 (0.10)	14.59 (0.31)	0.260	13.82 (0.10)	14.45 (0.38)	0.096
**Current smoker (%)**	48.8 (1.4)	43.0 (5.5)	0.312	6.7 (0.6)	9.7 (3.3)	0.283
**Heavy drinker (%)**	24.2 (1.4)	19.9 (4.4)	0.356	5.4 (0.5)	10.8 (3.4)	0.035
**Regular physical exerciser (%)**	24.4 (1.3)	24.6 (5.3)	0.967	19.0 (1.0)	17.1 (4.0)	0.668
**Hypertension (%)**	20.8 (1.1)	31.5 (4.6)	0.011	17.1 (0.8)	35.8 (5.5)	<0.001
**Diabetes (%)**	6.9 (0.7)	12.3 (3.3)	0.053	5.4 (0.5)	14.3 (4.0)	0.001
**Post-menopause (%)**				33.0 (1.2)	66.9 (5.8)	<0.001
**Total cholesterol (mg/dL)**	187.92 (1.05)	188.33 (5.27)	0.939	185.13 (0.85)	194.36 (4.41)	0.041
**HDL cholesterol (mg/dL)**	49.92 (0.35)	48.49 (1.27)	0.272	56.07 (0.28)	54.61 (1.39)	0.302
**LDL cholesterol (mg/dL)**	113.01 (1.39)	109.04 (7.24)	0.590	109.42 (1.34)	109.62 (9.03)	0.982
**TG cholesterol (mg/dL)**	157.89 (3.88)	177.34 (28.56)	0.495	105.10 (1.68)	121.41 (7.57)	0.035

Data are expressed as weighted mean (SEs), frequency (%).

OAG, open angle glaucoma; SE, standard error; HDL, high density lipoprotein; LDL, low density lipoprotein; TG, Triglycerides

**Table 2:** Baseline anthropometric parameters of study subjects by gender.

Variables	Healthy men	OAG men	P	Healthy women	OAG women	P
**BMI (kg/m**^**2**^**)**	24.15 (0.09)	23.57 (0.25)	0.023	23.20 (0.09)	24.18 (0.39)	0.015
**Weight (kg)**	71.16 (0.33)	68.60 (0.87)	0.006	57.85 (0.22)	57.58 (0.98)	0.789
**Height (cm)**	171.54 (0.19)	170.44 (0.72)	0.128	157.96 (0.15)	154.34 (0.63)	<0.001
**WC (cm)**	84.17 (0.27)	84.04 (0.81)	0.870	77.42 (0.27)	80.61 (1.11)	0.005
**Fat mass (kg)**	16.36 (0.20)	15.34 (0.43)	0.020	19.56 (0.16)	19.40 (0.68)	0.817
**Lean body mass (kg)**	54.22 (0.21)	52.68 (0.67)	0.029	37.87 (0.13)	37.78 (0.46)	0.860
**Non-bone lean body mass (kg)**	51.58 (0.21)	50.10 (0.65)	0.030	35.81 (0.12)	35.90 (0.44)	0.848
**Bone mineral content (kg)**	2.64 (0.01)	2.58 (0.03)	0.079	2.05 (0.01)	1.88 (0.04)	<0.001
**ASM mass (kg)**	22.86 (0.11)	22.05 (0.34)	0.019	14.51 (0.06)	14.45 (0.19)	0.770
**Fat mass/weight**	0.23 (0.00)	0.22 (0.01)	0.408	0.33 (0.00)	0.33 (0.01)	0.630
**Lean body mass/weight**	0.77 (0.00)	0.77 (0.01)	0.462	0.66 (0.00)	0.66 (0.01)	0.588
**Non-bone lean body mass/weight**	0.73 (0.00)	0.73 (0.01)	0.502	0.62 (0.00)	0.63 (0.01)	0.326
**ASM mass/weight**	0.32 (0.00)	0.32 (0.00)	0.852	0.25 (0.00)	0.25 (0.00)	0.559
**Fat mass/BMI**	0.66 (0.01)	0.64 (0.01)	0.145	0.83 (0.00)	0.79 (0.02)	0.014
**Lean body mass/BMI**	2.26 (0.01)	2.24 (0.03)	0.523	1.65 (0.01)	1.58 (0.02)	<0.001
**Non-bone lean body mass/BMI**	2.15 (0.01)	2.13 (0.02)	0.512	1.56 (0.01)	1.50 (0.02)	0.001
**ASM mass/BMI**	0.95 (0.00)	0.94 (0.01)	0.352	0.63 (0.00)	0.61 (0.01)	0.001
**Fat mass/lean body mass**	0.30 (0.00)	0.29 (0.01)	0.356	0.52 (0.00)	0.51 (0.02)	0.704
**Fat mass/non-bone lean body mass**	0.32 (0.00)	0.31 (0.01)	0.370	0.54 (0.00)	0.54 (0.02)	0.600
**Fat mass/ASM mass**	0.72 (0.01)	0.70 (0.02)	0.643	1.35 (0.01)	1.34 (0.04)	0.732

Data are presented as weighted mean (SEs).

BMI, body mass index; WC, waist circumference; ASM, appendicular skeletal muscle

### Risk of OAG according to anthropometric parameters

In males, BMI was associated with OAG (ORs = 0.936 (0.880–0.997), P = 0.038). In females, height and fat mass/BMI ratio was associated with OAG (AOR = 0.951 (0.910–0.994), P = 0.026, AOR = 0.171 (0.030–0.980), P = 0.047, respectively Table 3).

**Table 3:** Relationship between anthropometric parameters and the development of open-angle glaucoma.

Variables	Male		Female	
	ORs (95% CIs)	P^†^	ORs (95% CIs)	P^†^
**BMI (kg/m**^**2**^**)**				
Model 1	0.937 (0.882–0.996)	0.038	1.036 (0.966–1.111)	0.320
Model 2	0.936 (0.880–0.997)	0.038	1.032 (0.966–1.104)	0.350
**Weight (kg)**				
Model 1	0.989 (0.969–1.009)	0.274	0.998 (0.972–1.025)	0.910
Model 2	0.989 (0.969–1.009)	0.272	0.998 (0.973–1.024)	0.861
**Height (cm)**				
Model 1	1.021 (0.978–1.066)	0.349	0.952 (0.912–0.994)	0.026
Model 2	1.021 (0.977–1.066)	0.353	0.951 (0.910–0.994)	0.026
**WC (cm)**				
Model 1	0.984 (0.962–1.006)	0.154	1.005 (0.979–1.032)	0.692
Model 2	0.984 (0.962–1.007)	0.165	1.003 (0.978–1.029)	0.804
**Fat mass (kg)**				
Model 1	0.971 (0.941–1.003)	0.075	0.986 (0.940–1.035)	0.572
Model 2	0.970 (0.939–1.003)	0.073	0.986 (0.942–1.033)	0.554
**Lean body mass (kg)**				
Model 1	0.992 (0.956–1.030)	0.690	1.016 (0.970–1.064)	0.509
Model 2	0.992 (0.955–1.031)	0.698	1.014 (0.968–1.062)	0.556
**Non-bone lean body mass (kg)**				
Model 1	0.993 (0.955–1.032)	0.707	1.019 (0.972–1.069)	0.436
Model 2	0.993 (0.954–1.033)	0.716	1.017 (0.970–1.067)	0.479
**Bone mineral content (kg)**				
Model 1	0.815 (0.483–1.375)	0.441	0.632 (0.237–1.684)	0.357
Model 2	0.809 (0.480–1.364)	0.426	0.620 (0.231–1.662)	0.341
**ASM mass (kg)**				
Model 1	0.998 (0.925–1.077)	0.962	1.039 (0.948–1.138)	0.416
Model 2	0.997 (0.924–1.077)	0.945	1.038 (0.947–1.138)	0.420
**Fat mass/weight**				
Model 1	0.097 (0.003–2.839)	0.175	0.050 (0.001–4.161)	0.184
Model 2	0.089 (0.002–3.195)	0.185	0.059 (0.001–5.136)	0.214
**Lean body mass/weight**				
Model 1	9.407 (0.317–279.154)	0.194	23.575 (0.307–1809.978)	0.153
Model 2	10.170 (0.279–370.931)	0.206	21.085 (0.264–1685.917)	0.172
**Non-bone lean body mass/weight**				
Model 1	11.043 (0.299–407.830)	0.191	35.200 (0.434–2854.114)	0.112
Model 2	12.087 (0.258–566.258)	0.204	31.411 (0.367–2689.388)	0.129
**ASM mass/weight**				
Model 1	391.266 (0.119–1289574.511)	0.148	1998.780 (0.458–8714593.320)	0.075
Model 2	384.499 (0.085–1737490.397)	0.165	2329.672 (0.467–11610936.600)	0.074
**Fat mass/BMI**				
Model 1	0.561 (0.188–1.668)	0.297	0.161 (0.029–0.892)	0.037
Model 2	0.546 (0.172–1.734)	0.304	0.171 (0.030–0.980)	0.047
**Lean body mass/BMI**				
Model 1	2.307 (0.823–6.468)	0.112	0.749 (0.175–3.199)	0.695
Model 2	2.318 (0.813–6.613)	0.116	0.746 (0.176–3.167)	0.690
**Non-bone lean body mass/BMI**				
Model 1	2.448 (0.816–7.342)	0.110	0.804 (0.181–3.571)	0.774
Model 2	2.464 (0.805–7.543)	0.114	0.800 (0.181–3.536)	0.768
**ASM mass/BMI**				
Model 1	7.135 (0.799–63.712)	0.078	0.890 (0.053–14.864)	0.935
Model 2	6.957 (0.764–63.316)	0.085	0.966 (0.058–16.071)	0.981
**Fat mass/lean body mass**				
Model 1	0.226 (0.029–1.780)	0.157	0.294 (0.039–2.211)	0.233
Model 2	0.213 (0.024–1.871)	0.163	0.307 (0.041–2.306)	0.251
**Fat mass/non-bone lean body mass**				
Model 1	0.243 (0.034–1.751)	0.160	0.300 (0.045–2.001)	0.213
Model 2	0.230 (0.029–1.839)	0.166	0.313 (0.047–2.084)	0.229
**Fat mass/ASM mass**				
Model 1	0.535 (0.220–1.299)	0.166	0.620 (0.302–1.271)	0.190
Model 2	0.526 (0.207–1.335)	0.176	0.623 (0.304–1.279)	0.197

Data are presented as weighted mean (SEs) or weighted β-coefficient value [95% confidence intervals (CIs)].

^†^ Logistic Regression Analysis.

Model 1: adjusted for age, diabetes, hypertension and intraocular pressure

Model 2: adjusted for age, diabetes, hypertension, intraocular pressure, heavy drinking, current smoking and regular physical exercise.

BMI, body mass index; WC, waist circumference; ASM, appendicular skeletal muscle

### Risk of OAG according to anthropometric parameter quartiles

Table 4 shows the ORs (95% CIs) for OAG risk according to anthropometric parameter quartiles in males, focusing on statistically significant values. BMI-related parameters exhibited a significant OR for OAG males (P <0.05). BMI was negatively related to muscle mass parameters such as lean body mass/BMI, non-bone lean body mass/BMI, while ASM mass/BMI ratio was positively associated with BMI. Fat mass/non-bone lean body mass and fat mass/ASM mass ratio were negatively associated with OAG.

**Table 4:** Relationship between anthropometric parameter quartiles and the development of open-angle glaucoma in men.

Variables	Quartile to Anthropometric measurements in men				P^†^
	Quartile 1	Quartile 2	Quartile 3	Quartile 4	
**BMI (kg/m**^**2**^**)**					
Model 1	1.000	1.089 (0.589–2.013)	0.632 (0.362–1.106)	0.592 (0.303–1.156)	0.031
Model 2	1.000	1.085 (0.589–2.000)	0.618 (0.361–1.057)	0.591 (0.303–1.155)	0.028
**Weight (kg)**					
Model 1	1.000	1.286 (0.701–2.359)	0.979 (0.512–1.871)	0.951 (0.522–1.730)	0.651
Model 2	1.000	1.278 (0.694–2.353)	0.971 (0.509–1.851)	0.949 (0.517–1.742)	0.645
**Height (cm)**					
Model 1	1.000	0.817 (0.416–1.607)	1.836 (1.003–3.363)*	1.376(0.684–2.770)	0.100
Model 2	1.000	0.820 (0.417–1.612)	1.845 (1.017–3.346)*	1.369 (0.673–2.782)	0.106
**WC (cm)**					
Model 1	1.000	1.004 (0.514–1.960)	0.585 (0.311–1.102)	0.740 (0.416–1.318)	0.135
Model 2	1.000	1.002 (0.515–1.950)	0.584 (0.316–1.078)	0.741 (0.415–1.321)	0.139
**Fat mass (kg)**					
Model 1	1.000	1.515 (0.855–2.685)	0.614 (0.333–1.135)	0.892 (0.495–1.607)	0.154
Model 2	1.000	1.505 (0.837–2.707)	0.605 (0.327–1.122)	0.886 (0.490–1.604)	0.149
**Lean body mass (kg)**					
Model 1	1.000	1.365 (0.764–2.438)	1.002 (0.520–1.930)	1.170 (0.612–2.234)	0.912
Model 2	1.000	1.364 (0.765–2.432)	1.002 (0.521–1.928)	1.175 (0.609–2.264)	0.906
**Non-bone lean body mass (kg)**					
Model 1	1.000	1.368 (0.758–2.466)	1.002 (0.525–1.911)	1.188 (0.626–2.257)	0.873
Model 2	1.000	1.365 (0.758–2.456)	1.000 (0.525–1.905)	1.193 (0.623–2.285)	0.869
**Bone mineral content (kg)**					
Model 1	1.000	1.151 (0.618–2.145)	1.169 (0.673–2.031)	0.837 (0.461–1.517)	0.619
Model 2	1.000	1.146 (0.610–2.151)	1.165 (0.673–2.015)	0.830 (0.457–1.505)	0.603
**ASM mass (kg)**					
Model 1	1.000	1.438 (0.755–2.738)	1.587 (0.808–3.119)	1.172 (0.583–2.358)	0.616
Model 2	1.000	1.443 (0.759–2.745)	1.590 (0.802–3.153)	1.170 (0.577–2.370)	0.625
**Fat mass/weight**					
Model 1	1.000	0.649 (0.325–1.295)	0.730 (0.415–1.285)	0.469 (0.257–0.857)*	0.027
Model 2	1.000	0.644 (0.310–1.335)	0.723 (0.404–1.295)	0.460 (0.245–0.863)*	0.028
**Lean body mass/weight**					
Model 1	1.000	1.323 (0.669–2.616)	1.865 (0.948–3.671)	1.601 (0.837–3.065)	0.072
Model 2	1.000	1.331 (0.670–2.644)	1.885 (0.960–3.703)	1.615 (0.824–3.165)	0.073
**Non-bone lean body mass/weight**					
Model 1	1.000	1.538 (0.796–2.972)	1.791 (0.896–3.580)	1.683 (0.883–3.207)	0.091
Model 2	1.000	1.551 (0.797–3.019)	1.809 (0.910–3.597)	1.699 (0.877–3.293)	0.092
**ASM mass/weight**					
Model 1	1.000	1.136 (0.609–2.118)	1.667 (0.842–3.299)	1.506 (0.765–2.963)	0.130
Model 2	1.000	1.136 (0.608–2.123)	1.660 (0.847–3.256)	1.508 (0.745–3.050)	0.143
**Fat mass/BMI**					
Model 1	1.000	1.485 (0.874–2.521)	1.051 (0.534–2.069)	0.908 (0.487–1.694)	0.463
Model 2	1.000	1.476 (0.869–2.506)	1.046 (0.511–2.144)	0.904 (0.481–1.698)	0.475
**Lean body mass/BMI**					
Model 1	1.000	1.034 (0.515–2.077)	1.787 (0.922–3.464)	1.913 (1.000–3.658)*	0.017
Model 2	1.000	1.038 (0.521–2.066)	1.807 (0.926–3.527)	1.929 (1.007–3.694)*	0.016
**Non-bone lean body mass/BMI**					
Model 1	1.000	0.969 (0.484–1.943)	2.063 (1.089–3.910)*	1.556 (0.781–3.101)	0.043
Model 2	1.000	0.973 (0.489–1.938)	2.083 (1.100–3.947)*	1.567 (0.772–3.179)	0.046
**ASM mass/BMI**					
Model 1	1.000	0.878 (0.443–1.741)	2.072 (1.086–3.954)*	1.707 (0.894–3.261)	0.016
Model 2	1.000	0.879 (0.444–1.738)	2.069 (1.080–3.960)*	1.706 (0.893–3.260)	0.017
**Fat mass/lean body mass**					
Model 1	1.000	0.917 (0.467–1.800)	0.827 (0.462–1.480)	0.562 (0.307–1.028)	0.063
Model 2	1.000	0.913 (0.450–1.853)	0.820 (0.451–1.492)	0.554 (0.296–1.035)	0.063
**Fat mass/non-bone lean body mass**					
Model 1	1.000	0.918 (0.468–1.802)	0.887 (0.498–1.581)	0.498 (0.268–0.926)*	0.038
Model 2	1.000	0.917 (0.454–1.852)	0.882 (0.488–1.595)	0.490 (0.258–0.930)*	0.038
**Fat mass/ ASM mass**					
Model 1	1.000	0.970 (0.516–1.822)	0.727 (0.395–1.339)	0.510 (0.274–0.946)*	0.020
Model 2	1.000	0.961 (0.497–1.855)	0.719 (0.382–1.355)	0.504 (0.264–0.959)*	0.023

Data are presented as weighted means (SEs) or weighted β-coefficients [95% confidence intervals (CIs)].

^†^ P value for linear trend obtained by logistic regression analysis.

Model 1: adjusted for age, diabetes, hypertension, intraocular pressure

Model 2: adjusted for age, diabetes, hypertension, intraocular pressure, heavy drinking, current smoking, and regular physical exercise.

* p<0.05.

BMI, body mass index; WC, waist circumference; ASM, appendicular skeletal muscle.

Table 5 shows the ORs (95% CIs) for OAG risk according to anthropometric parameter quartiles in females. However, in females, only fat mass/BMI showed a significant relationship with the risk of OAG. A multivariate general linear model showed ORs were 0.703 (0.363–1.360) in quartile 2, 0.375 (0.175–0.801) in quartile 3, and 0.611 (0.341–1.093) in quartile 4 compared with quartile 1 (P = 0.040).

**Table 5:** Relationship between anthropometric parameter quartiles and the development of open-angle glaucoma in women.

Variables	Quartile to Anthropometric measurements in men				P^†^
	Quartile 1	Quartile 2	Quartile 3	Quartile 4	
**BMI (kg/m**^**2**^**)**					
Model 1	1.000	0.853 (0.406–1.793)	0.508 (0.222–1.161)	1.103 (0.555–2.192)	0.824
Model 2	1.000	0.886 (0.413–1.902)	0.519 (0.225–1.197)	1.116 (0.554–2.251)	0.816
**Weight (kg)**					
Model 1	1.000	0.693 (0.371–1.295)	0.631 (0.316–1.259)	0.716 (0.407–1.261)	0.255
Model 2	1.000	0.708 (0.371–1.351)	0.648 (0.315–1.336)	0.724 (0.406–1.289)	0.286
**Height (cm)**					
Model 1	1.000	0.646 (0.356–1.170)	0.446 (0.213–0.933)*	0.520 (0.236–1.142)	0.030
Model 2	1.000	0.640 (0.353–1.160)	0.438 (0.209–0.919)*	0.520 (0.233–1.158)	0.031
**WC (cm)**					
Model 1	1.000	1.110 (0.612–2.013)	0.646 (0.318–1.310)	1.108 (0.579–2.121)	0.997
Model 2	1.000	1.069 (0.582–1.963)	0.655 (0.318–1.347)	1.070 (0.572–2.001)	0.971
**Fat mass (kg)**					
Model 1	1.000	0.551 (0.272–1.118)	0.491 (0.236–1.020)	0.751 (0.412–1.370)	0.393
Model 2	1.000	0.562 (0.275–1.146)	0.509 (0.240–1.080)	0.756 (0.413–1.386)	0.418
**Lean body mass (kg)**					
Model 1	1.000	0.917 (0.435–1.934)	0.671 (0.322–1.399)	1.347 (0.762–2.383)	0.450
Model 2	1.000	0.939 (0.440–2.004)	0.703 (0.334–1.482)	1.348 (0.742–2.448)	0.458
**Non-bone lean body mass (kg)**					
Model 1	1.000	1.047 (0.507–2.163)	0.501 (0.231–1.086)	1.478 (0.847–2.578)	0.399
Model 2	1.000	1.091 (0.521–2.284)	0.528 (0.242–1.155)	1.497 (0.832–2.695)	0.401
**Bone mineral content (kg)**					
Model 1	1.000	0.935 (0.489–1.788)	0.618 (0.292–1.308)	0.794 (0.317–1.990)	0.465
Model 2	1.000	0.904 (0.465–1.759)	0.602 (0.283–1.281)	0.749 (0.294–1.908)	0.404
**ASM mass (kg)**					
Model 1	1.000	1.239 (0.621–2.474)	1.220 (0.650–2.291)	1.311 (0.659–2.611)	0.459
Model 2	1.000	1.253 (0.625–2.514)	1.257 (0.664–2.381)	1.332 (0.666–2.663)	0.425
**Fat mass/weight**					
Model 1	1.000	0.834 (0.424–1.639)	0.663 (0.311–1.412)	0.756 (0.401–1.422)	0.325
Model 2	1.000	0.823 (0.420–1.613)	0.669 (0.314–1.427)	0.760 (0.402–1.438)	0.349
**Lean body mass/weight**					
Model 1	1.000	0.920 (0.460–1.836)	1.175 (0.567–2.433)	1.529 (0.813–2.877)	0.159
Model 2	1.000	0.930 (0.464–1.863)	1.193 (0.576–2.473)	1.529 (0.800–2.922)	0.165
**Non-bone lean body mass/weight**					
Model 1	1.000	0.801 (0.389–1.647)	0.948 (0.464–1.938)	1.619 (0.867–3.025)	0.127
Model 2	1.000	0.815 (0.396–1.677)	0.943 (0.462–1.924)	1.629 (0.863–3.075)	0.134
**ASM mass/weight**					
Model 1	1.000	0.691 (0.308–1.551)	1.407 (0.708–2.798)	1.413 (0.738–2.709)	0.119
Model 2	1.000	0.725 (0.325–1.618)	1.413 (0.713–2.800)	1.454 (0.759–2.786)	0.110
**Fat mass/BMI**					
Model 1	1.000	0.709 (0.369–1.362)	0.380 (0.181–0.800)*	0.618 (0.344–1.111)	0.042
Model 2	1.000	0.703 (0.363–1.360)	0.375 (0.175–0.801)*	0.611 (0.341–1.093)	0.040
**Lean body mass/BMI**					
Model 1	1.000	1.058 (0.565–1.981)	0.766 (0.362–1.621)	1.168 (0.559–2.441)	0.971
Model 2	1.000	1.067 (0.571–1.993)	0.770 (0.368–1.611)	1.174 (0.563–2.448)	0.963
**Non-bone lean body mass/BMI**					
Model 1	1.000	1.035 (0.549–1.950)	0.816 (0.383–1.738)	1.270 (0.613–2.632)	0.728
Model 2	1.000	1.035 (0.550–1.948)	0.825 (0.392–1.736)	1.275 (0.615–2.642)	0.717
**ASM mass/BMI**					
Model 1	1.000	1.146 (0.595–2.205)	0.693 (0.319–1.509)	1.183 (0.594–2.357)	0.970
Model 2	1.000	1.144 (0.596–2.193)	0.678 (0.312–1.470)	1.193 (0.593–2.399)	0.958
**Fat mass/lean body mass**					
Model 1	1.000	0.737 (0.367–1.479)	0.633 (0.303–1.322)	0.697 (0.372–1.306)	0.255
Model 2	1.000	0.739 (0.368–1.483)	0.645 (0.308–1.349)	0.703 (0.371–1.332)	0.277
**Fat mass/non-bone lean body mass**					
Model 1	1.000	0.746 (0.375–1.483)	0.638 (0.309–1.316)	0.687 (0.365–1.293)	0.232
Model 2	1.000	0.741 (0.372–1.473)	0.651 (0.314–1.348)	0.688 (0.362–1.309)	0.250
**Fat mass/ ASM mass**					
Model 1	1.000	0.875 (0.448–1.712)	0.487 (0.232–1.020)	0.728 (0.384–1.379)	0.181
Model 2	1.000	0.869 (0.445–1.699)	0.506 (0.239–1.072)	0.710 (0.377–1.336)	0.169

Data are presented as weighted means (SEs) or weighted β-coefficients [95% confidence intervals (CIs].

^†^ P value for linear trend obtained by logistic regression analysis.

Model 1: adjusted for age, diabetes, hypertension, intraocular pressure

Model 2: adjusted for age, diabetes, hypertension, intraocular pressure, heavy drinking, current smoking, and regular physical exercise.

* p<0.05.

BMI, body mass index; WC, waist circumference; ASM, appendicular skeletal muscle.

## Discussion

Glaucoma has been associated with various independent risk factors that include IOP-dependent and IOP-independent mechanisms. IOP is now thought to be the most important risk factor for glaucoma, even though glaucoma severity is not always directly correlated with increased IOP. Consequently, researchers have looked for other contributing factors to explain glaucoma pathogenesis, such as genetics, vascular dysregulation and oxidative stress [22,23].

Not only fat tissue, but also muscle contribute to a person’s weight, and people who are more active might develop more muscle mass, thus adding to their weight. BMI alone cannot determine exact body composition. Therefore, BMI as the most commonly used anthropometric index to define obesity is currently controversial, due to its inability to discriminate between fat and muscle mass [24]. Therefore we aimed to elucidate possible differential effects of fat and muscle ratio as well as weight or BMI as risk factors for open angle glaucoma by gender.[25]

Considering the difference in body composition between males and females, a difference in anthropometric parameter effects should be expected. According to our results, in terms of the relationship with OAG, female body composition was more affected by fat tissue and males by muscle. Men were more vulnerable in terms of anthropometric parameter association with OAG. In females, higher fat mass was associated with lower OAG prevalence. In males, higher muscle mass was positively correlated with OAG. In males, BMI showed an inverse association with muscle mass parameters (lean body mass, non-bone lean body mass, and ASM mass). Glaucoma risk was also negatively associated with BMI. More muscle for a given BMI was associated with higher OAG risk and higher fat for a given muscle mass was associated with lower risk for OAG. Specifically, interquartile analysis showed that for muscle/BMI, the change in OR was minimal in quartile 2 but abruptly increased in quartile 3. Counterintuitively, increased muscle mass might not be advantageous in terms of OAG. These associations persisted after adjusting for several demographic parameters and IOP, suggesting that certain anthropometric parameters significantly affect the development of OAG through pathways independent of blood pressure, diabetes, or IOP.

Some reports have suggested that glaucoma was associated with anthropometric parameters. Several studies have found an association,[3,10,26] while others have not [4,5]. Casey et al. [3] reported that obese Caucasian females had a 6% increased risk of developing OAG compared with non-obese females. However, Gasser et al. reported that BMI tended to be lower in patients with glaucoma than in control subjects, but this difference was not significant. They suggested that obesity was not associated with glaucoma [5]. The Tajimi study [27] reported similar results. In the Barbados Eye Study, high IOP and lean body mass but not obesity were significantly associated with glaucoma [4]. Pasquale et al. [10] reported that higher BMI was associated with a lower risk of normal tension glaucoma in females. These previous studies obtained varied results due to differences in study design, population, and definitions of obesity and glaucoma.

To date, the causal relationship between body composition and development of OAG has remained unclear. A hypothesis on the relationship between obesity and OAG development is that hyperleptinemia accompanying obesity leads to increased oxidative stress [28,29]. Several studies have shown that patients with OAG have higher oxidative damage in their trabecular meshwork [30,31]; reportedly, oxidative stress affects glaucomatous optic neuropathy [32]. Additionally, females have higher leptin levels than males [33].

Another factor may be the relationship between cerebrospinal fluid pressure (CSFP) and body mass [34,35]. Considering the interfacial lamina cribrosa (LC) membrane location, increased translaminar pressure by elevated IOP and/or reduced CSFP might induce the ganglion cell axon loss seen in glaucoma. In terms of LC backward bowing due to a high pressure gradient, lower CSFP may act similarly to elevated IOP and increase the risk for glaucomatous damage. Several studies have found that CSFP is lower in OAG patients and increased in ocular hypertension patients [34–36]. According to previous reports, BMI has been positively associated with CSFP [37–39]. In view of this situation, slightly elevated CSFP would reduce the translaminar pressure difference and might play a protective role against glaucoma development.

Shah et al. reported cases of exercise-related visual loss in advanced glaucoma patients who were young adults with healthy visual acuity but presented with visual field constriction upon relatively mild exertion [40]. They hypothesized that ocular blood flow was reduced in these patients during exercise as blood was diverted to other organs: a “vascular steal hypothesis.” Activities such as eating and drinking could cause a 'vascular steal,' with blood from the ocular circulation being shunted to the skin and stomach muscles. Considering this hypothesis, the vascular steal phenomenon may be more prominent in persons who have a large muscle mass compared to those with smaller muscle mass during exercise or activities of daily living.

Naturally, BMI increases throughout life until 60–70 years of age [41–44]. A long-recognized age-related phenomenon is the gradual increase in visceral fat and aging-related loss of skeletal muscle mass (sarcopenia) with associated changes in muscle quality and function [45]. Skeletal muscle mass reaches its peak during the late teen years and early 20s and then slowly declines in healthy adults at a rate of approximately 0.5–1% per year, which is a natural course and might be an aging-related adaptation. Age-related rates of skeletal muscle mass loss vary according to individual. Muscle is a dynamic energy consumption tissue, while fat is a static storage tissue. Skeletal muscle comprising up to 40–50% of the total body mass is a determinant of oxygen consumption, whole body energy metabolism, and substrate turnover and storage. Generally increased muscle mass and decreased adipose tissue are regarded as positive effects on health, especially metabolic syndrome. However, the opposite occurs in some cases, which is referred to as the ‘obesity paradox’ [46]. Large muscle mass can be beneficial against certain diseases or to certain individuals but can affect the optic nerve differently. One report has suggested that glaucoma prevalence was not less than expected in higher performance athletes but might be higher than normal for subjects in poor health [47,48]. Therefore, the authors suggested that physical exercise might not be beneficial with regard to glaucoma pathology [49]. To determine the exact pathophysiology, further studies are needed.

The present study had several limitations. First, this study was a cross-sectional design and so was limited in detecting causal relationships. Therefore, cause-and-effect relationships between anthropometric parameters and OAG cannot be inferred. Second, we performed an FDT matrix test to evaluate visual fields, which is not standard automated perimetry. Third, due to the limited epidemiological study setting of KNHANES, we could not evaluate gonioscopy data, which is the gold standard for determining angle status. Instead, we used peripheral ACD > 1/4 corneal thickness as a definition of open angle based on Van-Herick’s method. However, we used the data from KHANES V, which subdivided ocular surgeries into glaucoma, cataract, retinal, refractive, strabismus, eyelid surgery, and others, so that we could enroll subjects more properly. Additionally, KNHANES subjects were enrolled after required visits, resulting in a possible selection bias because severely ill patients suffering from cachexia or extremely obese subjects who were either institutionalized or had ambulatory difficulties could not participate. Lastly, the effects of anthropometric parameters based on gender differences are unexplained but might be due to basic differences in quality and quantity of fat and muscle according to original differences in body composition. We think, based on our subdivided analyses of body compositions, a more customized prognosis could be provided.

Glaucomatous damage may be associated with various contributing etiological factors and some may be related to IOP level more than others [50].

In conclusion, the present study evaluated not only the effect of obesity, but the effects of fat and muscle on OAG development. In terms of glaucoma development, our results indicated that fat seemed protective while muscle was not. Obesity is not a risk factor for OAG development. The exact mechanism remains unclear, and longitudinal studies are needed to validate and extend our findings.
